# The effects of vildagliptin on glycemic variability in patients with type 2 diabetes on premixed insulin therapy

**DOI:** 10.3389/fendo.2025.1508918

**Published:** 2025-06-20

**Authors:** Deyue Kong, Ziyang Shen, Lanlan Jiang, Xiaojing Xie, Rengna Yan, Ting Jing, Yun Hu, Jianhua Ma

**Affiliations:** ^1^ Department of Endocrinology, Nanjing First Hospital, Nanjing Medical University, Nanjing, China; ^2^ Department of Endocrinology, the Affiliated Wuxi People’s Hospital of Nanjing Medical University, Wuxi People’s Hospital, Wuxi Medical Center, Nanjing Medical University, Wuxi, China

**Keywords:** vildagliptin, type 2 diabetes mellitus, glycemic excursion, flash glucose monitoring, premixed insulin

## Abstract

**Aim:**

Patients on premixed insulin therapy usually have poor glycemic control. This study aimed to investigate the effect of vildagliptin in these patients.

**Methods:**

This real-world study included patients with type 2 diabetes mellitus (T2DM), who were poorly glycemic controlled on premixed insulin therapy and were subsequently added vildagliptin. The control group consisted of patients who only had their insulin doses adjusted without adding vildagliptin, matched for age, diabetic duration, HbA1c, and BMI. All patients underwent FGM, glycated hemoglobin(HbA1c), and glycated albumin(GA) measurements at baseline and three months after the treatment adjustment.

**Results:**

Patients receiving vildagliptin treatment demonstrated significant reductions in HbA1c and GA levels (P<0.001 and P=0.009, respectively). The vildagliptin group exhibited a remarkable decrease in the mean amplitude of glycemic excursion (MAGE) (8.58 ± 0.36 *vs*. 6.62 ± 0.47, P<0.001), along with notable reductions in mean blood glucose (MBG) (10.7 ± 0.34 *vs*. 8.82 ± 0.39, P<0.001) and time above the target range (TAR) (52.60 ± 3.44 *vs*. 31.59 ± 4.31, P<0.001) compared to the control group. Moreover, there were notable improvements in the duration spent within the target range (TIR) (45.64 ± 3.33 *vs*. 64.22 ± 4.00, P<0.001), along with increases in the areas under the curve (AUC) for blood glucose levels above 4.4 (426.82 ± 83.19 *vs*. 892.16 ± 185.27, P=0.018) and 3.9 (213.81 ± 47.20 *vs*. 454.77 ± 103.21, P=0.029). Hourly mean blood glucose levels over a 2-week period monitored by FGM indicated lower blood glucose levels in the vildagliptin group, particularly after dinner (P=0.022).

**Conclusion:**

Vildagliptin added to premixed insulin effectively lowers blood glucose levels and reduces glycemic variability in patients with type 2 diabetes mellitus.

**Clinical Trial Registration:**

https://clinicaltrials.gov, identifier NCT04847219.

## 1 Introduction

Type 2 diabetes mellitus (T2DM) has become increasingly prevalent worldwide, emerging as a major public health concern (1). The disease is characterized by sustained hyperglycemia, often accompanied by pancreatic β-cell dysfunction, insulin resistance, and immune inflammation, which collectively contribute to its progression (2). Disruptions in multiple metabolic pathways play a crucial role in the development of T2DM complications, which are major drivers of morbidity and mortality (3). The increasing prevalence of T2DM, along with its long-term complications, imposes a substantial economic burden on healthcare systems worldwide (4).

As the disease progresses, in many cases patients with T2DM need to be treated with insulin to help them better achieve their intended therapeutic goals (5). This indicates the necessity of insulin regimens that focus on strict postprandial glucose control. As a result, premixed insulin has become a widely used option for initiating insulin therapy in China due to its greater convenience in managing both basal and prandial glucose (6). Premixed insulin combines both basal and prandial insulin, and while this simplifies the dosing regimen, it leads to less flexibility in managing glucose levels. This reduced flexibility can result in a mismatch between insulin action and meal timing, increasing the likelihood of both mild and severe hypoglycemia (7). Studies indicate that patients using premixed insulin tend to experience more frequent hypoglycemic events compared to those on basal-bolus regimens (8). Premixed insulin may also cause larger fluctuations in blood glucose levels. Its fixed ratio of rapid-acting and long-acting components makes it difficult to adjust insulin doses precisely to cover varying postprandial and fasting glucose needs (9).

As a result, patients on premixed insulin therapy often experience significant swings in blood glucose levels, which can increase the risk of complications. Large blood glucose variability may be a risk factor for stroke and heart disease in individuals with T2DM, regardless of their glycated hemoglobin (HbA1c) levels (10). Studies also indicate that glucose variability is associated with microvascular complications such as retinopathy, neuropathy, and nephropathy. These fluctuations lead to cellular damage by inducing excessive production of reactive oxygen species (ROS), exacerbating oxidative stress, and inflammation and activating harmful signaling pathways (11).Aggressive modification of the treatment regimen is needed to reduce the patient’s level of glycemic fluctuation and to reduce complications.

Dipeptidyl peptidase-4(DPP-4)inhibitors can effectively improve blood glucose control in individuals with T2DM and notably decrease glucose variability when compared to alternative oral medications for diabetes (12, 13). Vildagliptin is one of the drugs in the class of DPP-4 inhibitors (14). This DPP-4 inhibitor enhances islet cell function by promoting insulin secretion and suppressing glucagon release. Patients with T2DM have been observed to have altered levels of glucagon-like peptide-1 (GLP-1), which may contribute to glucose regulation (15, 16). As an adjunct therapy for patients with inadequately controlled type 2 diabetes, vildagliptin produces dose-dependent reductions in HbA1c and FPG (17). A systematic review also concluded that vildagliptin has a good safety profile and a relatively low risk of causing hypoglycemia compared with other diabetes medications (18). Vildagliptin improves blood glucose variability by reducing metrics such as the Mean Amplitude of Glycemic Excursions (MAGE). Therefore, the efficacy and safety of the combination of vildagliptin with premixed insulin remains unclear.

Therefore, we conducted this real-world study to assess the effect of vildagliptin on glycemic variability using FGM before and after a 90-day treatment in patients on premixed insulin therapy.

## 2 Methods

### 2.1 Subjects

This research project is a secondary analysis of a premixed insulin study, which was conducted in the outpatients from the department of Endocrinology of five hospitals in Jiangsu Province from October 2019 to April 2021(ClinicalTrials.gov NCT04847219). The study was approved by the ethics committee of Nanjing First Hospital before it was conducted. All operations were in accordance with the ethical standards of the hospital and the 1964 Helsinki Declaration revised in 2013. Every patient submitted signed consent forms to participate in this study.

The inclusion criteria were as follows: 1)type 2 diabetes patients who met WHO1999 diagnostic criteria injected premix insulin subcutaneously, used a single drug or a combination of oral hypoglycemic medications for more than two months were on a stable treatment regimen; 2)no acute complications associated with diabetes, such as ketoacidosis and hyperosmolar disease; 3)FGM examinations, diets, and exercises are readily accepted by the subjects.

Exclusion criteria included the following: 1)within the last 3 months, patients who have taken GLP-1 agonists; 2)patients with insulin allergies; 3)the ALT was 2.5 times higher than the upper limit of normal value, and the serum creatinine level was 1.3 times higher than the upper limit of normal; both liver and renal function were impaired; 4)an alcohol or drug abuse history within the last five years; 5)last 3 months have been treated with systemic hormones; 6)poorly compliant and irregularly exercising patients; 7)infected and stressed patients within four weeks; 8)patients who cannot tolerate flash glucose monitoring; 9)pregnant, nursing, or pregnant patients; 10)additional conditions or complications that the investigator determines to be present, including severe heart disease, endocrine disease, lung disease, tumor disease, neurological disease, past mental illness, etc.

Both the control and experimental groups were selected using identical inclusion and exclusion criteria.

### 2.2 Study design

**Figure 1:**
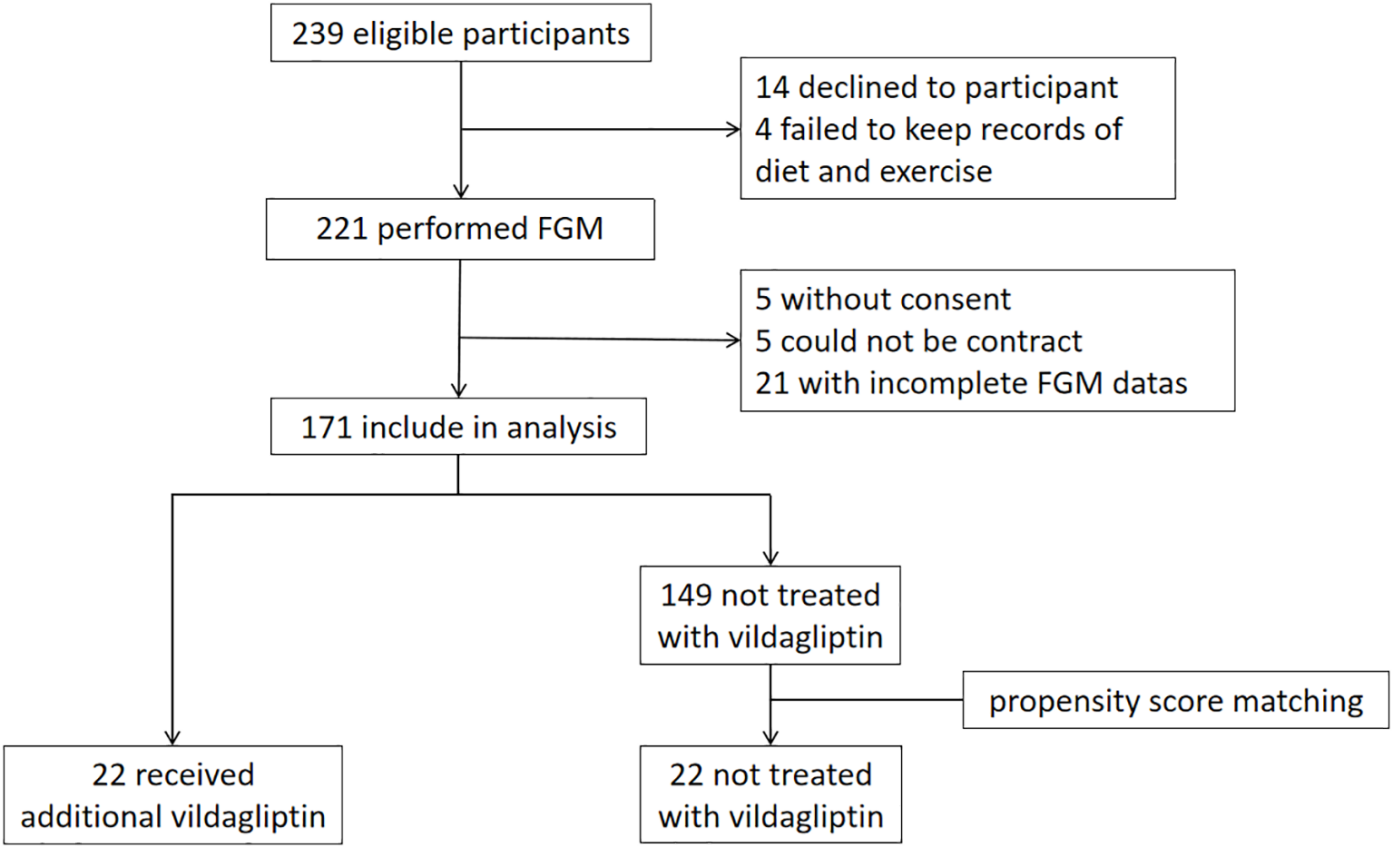
Trial profile.

**Figure 1** shows the outline of the study. Patients received FGM testing for 14 days at the beginning of the study. C-peptide, HbA1c, GA, glucagon and insulin levels were tested. Height, weight, and insulin dosage were recorded. Patients made adjustments to their insulin or oral medication regimen based on the results of FGM and the blood tests. Insulin doses were further adjusted during follow-up visits according to blood glucose levels, with FGM testing repeated after 3 months and subsequent reassessment of C-peptide levels, HbA1c, etc.

Based on this study, patients who received additional vildagliptin treatment during the premixed insulin study were chosen as the vildagliptin group (n=22). The control group was selected from the remaining patients who were not treated with vildagliptin through propensity score matching adjusted for age, duration of diabetes, HbA1c, BMI, and metformin dosage (n=22).

The blood glucose changes before and after treatment were compared between the two groups of patients. Glucagon and insulin concentrations in plasma were measured by radioimmunoassay, and C-peptide concentrations were measured by electrochemiluminescence. We measured HbA1c with high-performance liquid chromatography (BioRad, Diastat HbA1c analyzer) and glycated albumin (GA) with a peroxidase kit (Jiuqiang).

### 2.3 Statistical analysis

The statistical analysis was carried out utilizing SPSS23.0 software (SPSS, IL, USA). Data from normal distribution were expressed as mean ± standard error (SE), and data from nonnormal distribution were expressed as median in quartile ranges. The percentage of hypoglycemic drugs and the number of subjects with diabetes complications were analyzed using the chi-square test, and the data collected before and after treatment were evaluated using the Student paired t-test or Wilcoxon test. Hourly mean blood glucose concentrations assessed by two FGMs at baseline and endpoint were analyzed by Repeated Measures ANOVA with time as the within-subject factor and groups as the between-subject factor. The significance level was 5%.

## 3 Results

### 3.1 Baseline characteristics

In this study, 44 patients with T2DM were enrolled. 22 patients were enrolled in the vildagliptin group, and another 22 patients who did not take vildagliptin were matched as controls. Baseline characteristics included age, gender, weight, BMI, diabetic duration, insulin duration, HbA1c, GA, glucose-lowering drugs, and diabetic complications. At baseline, no significant differences were found between the two groups in terms of any of the characteristics (P all > 0.05, **Table 1**).

**Table 1:** Baseline characteristics of participants.

Baseline	Vildagliptin	Control	P value
N	22	22	
Age (yrs.)	59.95 ± 2.07	65.04 ± 1.77	0.068
Gender (male)	16 (72.73%)	13 (59.09%)	0.352
Weight (kg)	70.49 ± 1.89	67.41 ± 2.73	0.358
BMI (kg/m2)	25.09 ± 0.61	25.14 ± 0.67	0.953
Diabetic duration (month)	149.18 ± 18.55	167.45 ± 17.84	0.482
Insulin duration (month)	72 (36,111)	84 (33,150)	0.663
HbA1c (%)	8.17 ± 0.10	7.46 ± 0.15	0.699
GA (%)	21.13 (18.75,24.40)	20.21 (16.84,22.86)	0.585
Glucose-lowering drugs			
Insulin dose (IU/day)	40.45 ± 2.74	37.45 ± 2.25	0.403
Metformin (%)	9 (40.91%)	9 (40.91%)	0.863
Diabetic complications			
Diabetic kidney disease (%)	3 (13.64%)	3 (13.64%)	1.000
Neuropathy (%)	2 (9.09%)	5 (22.73%)	0.226
Retinopathy (%)	6 (27.27%)	3 (13.64%)	0.273
Coronary heart disease (%)	6 (27.27%)	4 (18.18%)	0.232
Cerebral infarction (%)	4 (18.18%)	3 (13.64%)	0.689
Fatty liver (%)	8 (36.36%)	14 (63.64%)	0.073

Data were presented as mean ± SE or median (25th, 75th percentile)or number (percentage). Difference between two groups with the Mann–Whitney U-test or chi-square test. BMI, body mass index; HbA1c, glycated hemoglobin; GA, Glycated Albumin.

### 3.2 Blood parameters

HbA1C levels significantly decreased after 3 months in both groups, while GA levels only showed a superior decrease in the vildagliptin group (P<0.05). No significant difference was found in the change of glucose, C-peptide, and insulin between the two groups (**Table 2**).

**Table 2:** Blood parameters.

Items	Vildagliptin (n=22)		P value	Control (n=22)		P value
	Before	After		Before	After	
HbA1c (%)	8.17 ± 0.10	7.46 ± 0.15	0.001	8.30 ± 0.30	7.75 ± 0.24	0.007
GA (%)	21.13 (18.75,24.40)	20.21 (16.84,22.86)	0.009	22.12 (17.81,23.84)	19.75 (17.71,23.96)	0.064
Glucagon(ng/L)	141.36 (124.14,157.94)	154.77 (129.48,168.20)	0.733	142.38 (129.11,167.04)	151.31 (126.41,171.47)	0.783
C-peptide (ng/ml)	1.23 (0.90,2.00)	1.30 (0.80,2.15)	0.673	1.46 (0.60,2.02)	1.21 (0.7,1.91)	0.263
Insulin (pmol/l)	15.25 (10.55,42.93)	12.5 (8.78,32.78)	0.3114	20.15 (9.95,55.28)	15.35 (8.08,56)	0.192

Data were presented as mean ± SE or median (25th, 75th percentile).

### 3.3 Glycemic control

After vildagliptin treatment, significant reductions were observed in mean blood glucose (MBG), MAGE, time above the target range (TAR)(P all <0.05), and significant increases in time in the target range (TIR), time below the target range (TBR), AUC(Area Under the Curve)>4.4, and AUC>3.9 after treatment as compared with those before treatment (P all <0.05) (**Table 3**).

**Table 3:** Changes in blood glycemic excursion parameters in the the vildagliptin and control groups before and after therapy.

Items	Vildagliptin (n=22)		P value	Control (n=22)		P value
	Before	After		Before	After	
MBG (mmol/L)	10.7 ± 0.34	8.82 ± 0.39*	0.001	10.26 ± 0.88	10.25 ± 0.81	0.982
MAGE (mmol/L)	8.58 ± 0.36	6.62 ± 0.47*	<0.001	7.91 ± 0.70	8.20 ± 0.78	0.609
CV(%)	33.57 ± 1.27	32.97 ± 1.34	0.647	33.19 ± 1.33	32.90 ± 1.80	0.853
TIR (%)	45.64 ± 3.33	64.22 ± 4.00*	0.001	52.35 ± 5.27	52.73 ± 5.58	0.941
TAR (%)	52.60 ± 3.44	31.59 ± 4.31*	<0.001	42.52 ± 6.00	43.41 ± 6.09	0.867
TBR (%)	1.76 ± 0.32	4.19 ± 1.08	0.033	5.13 ± 2.09	3.87 ± 1.35	0.432
AUC<4.4(mmol/L)	426.82 ± 83.19	892.16 ± 185.27	0.018	1345.43 ± 538.35	1039.91 ± 381.32	0.458
AUC<3.9(mmol/L)	213.81 ± 47.20	454.77 ± 103.21	0.029	717.61 ± 296.92	574.77 ± 238.14	0.544

MBG, mean blood glucose; MAGE, mean amplitude of glycemic excursion; CV, coefficient of variation; TIR, time in target range; TAR, time above target range; TBR,time below target range; AUC, Area Under Curve. AUC<4.4 incremental area under the curve of plasma glucose<4.4 mmol/L, AUC<3.9 incremental area under the curve of plasma glucose<3.9 mmol/L. *, Compared with the control group, P<0.05.

In addition, we compared the vildagliptin group with the control group after treatment using covariance analysis. In the vildagliptin group, MBG, MAGE, and TAR were significantly lower than in the control group at the end of treatment, while TIR showed a significant increase (P<0.05) (**Table 3**).

**Figure 2:**
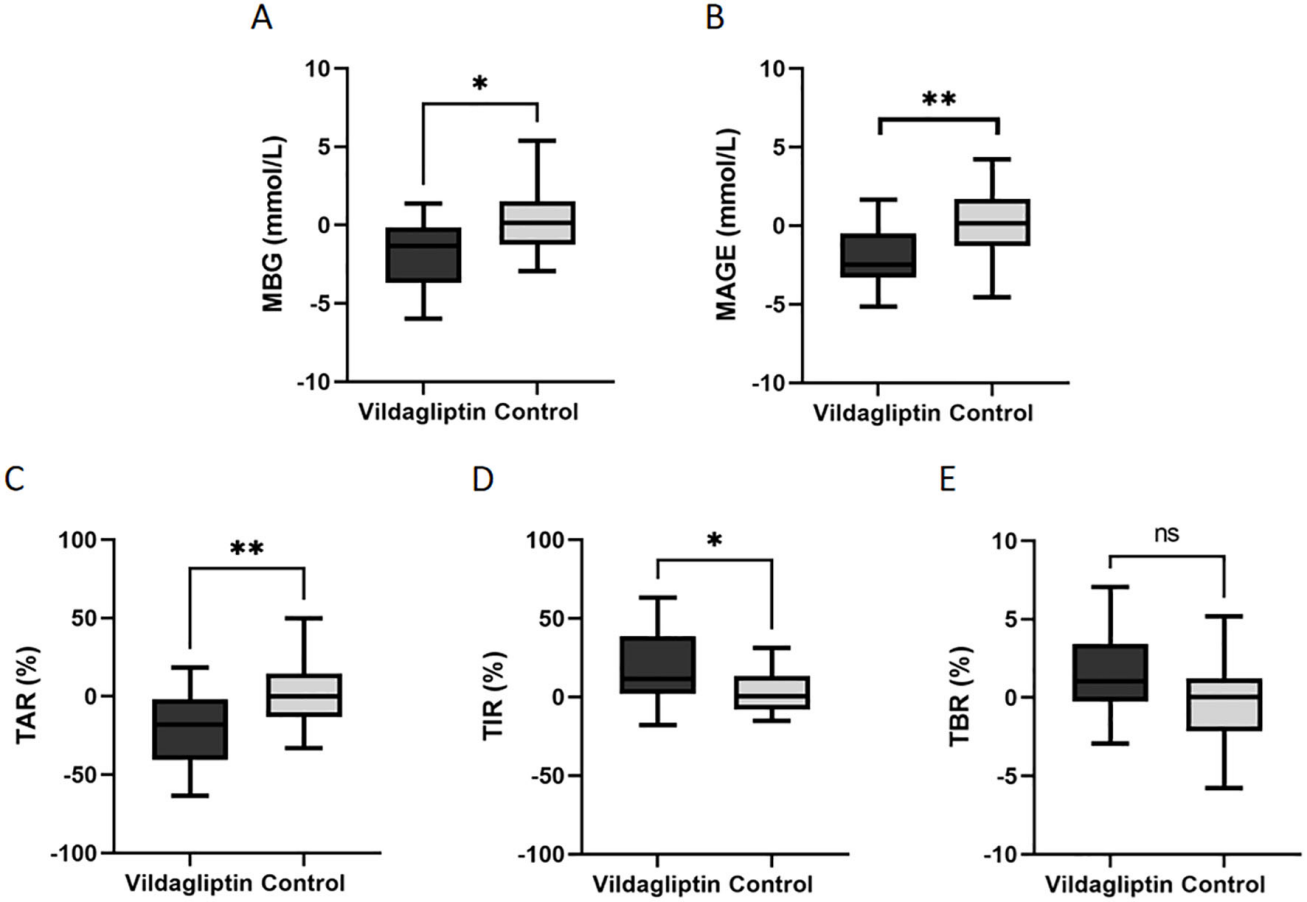
Changes in the levels of mean blood glucose(MBG), mean amplitude of glycemic excursion(MAGE), time above the target range(TAR), time in the target range (TIR) and time below the target range(TBR) between the vildagliptin and control groups. Data are mean ± SE. *, p < 0.05 between the two groups; **, p < 0.001 between the two groups. ns, not significant (P ≥ 0.05).

The change of blood glucose levels = blood glucose levels after treatment - blood glucose levels before treatment. Compared to the control group, patients taking vildagliptin showed superior reductions in MBG, MAGE and TAR (estimated treatment difference: -1.871 ± 0.734, -2.264 ± 0.75, -21.9 ± 7.223, P=0.015, 0.003 and 0.004, respectively, **Figure 2A-C**) and superior increase in TIR (estimated treatment difference:18.21 ± 6.971, P=0.012, **Figures 2D**). In either group, there were no significant differences in the change of TBR (**Figure 2E**).

### 3.4 The insulin dosage

Compared to the control group (30.45 ± 10.55*vs*.37.09 ± 11.30, P=0.603), patients in the vildagliptin group experienced a more significant reduction in daily insulin dosage before and after treatment (40.45 ± 1.86*vs*.36.68 ± 15.13, P=0.050). However, there was no significant difference in daily insulin dosage between the two groups after treatment(37.09 ± 11.30*vs*.36.68, P=0.705).

### 3.5 The change in hourly mean blood glucose concentrations

**Figure 3:**
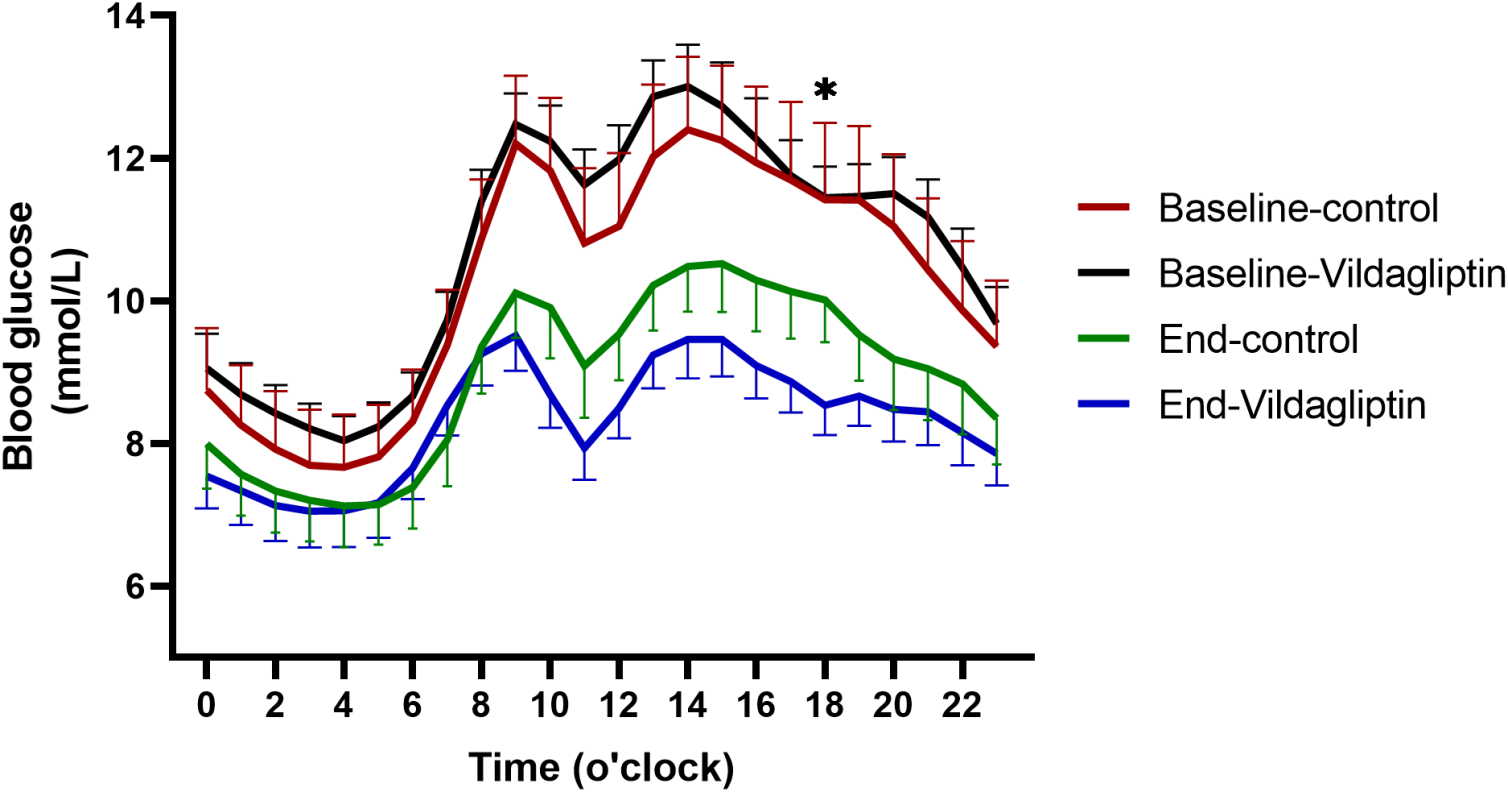
Hourly mean blood glucose during the first and second FGMs. Red solid line, first FGM in the control group(n=22); green solid line, second FGM in the control group; black solid line, first FGM in the vildagliptin group(n=22); blue solid line, second FGM in the vildagliptin group. *p < 0.05 between the end of two groups.

The vildagliptin group had significantly lower hourly mean blood glucose concentrations following 3 months of treatment than the control group (P=0.016, **Figure 3**). Participants who took vildagliptin after two 14-day FGM periods had lower blood glucose levels as measured by hourly mean blood glucose, especially after dinner (18:00) (8.53 ± 0.41*vs*.10.01 ± 0.59, P=0.022, **Figure 3**) which were similar between the two groups at baseline (P all >0.05). Nocturnal blood glucose changed similar (P all >0.05, **Figure 3**). Compared to the FGM at baseline, vildagliptin significantly decreased the hourly mean blood glucose during the second FGM (P=0.001).

## 4 Discussion

In this real-world study, it can be inferred that vildagliptin, acting as a DPP-4 inhibitor, demonstrates efficacy in lowering average blood glucose levels and diminishing the average magnitude of glycemic fluctuations in individuals.

Vildagliptin can prolong the half-life of incretin hormones such as glucagon-like peptide 1 (GLP-1) and glucose-dependent insulinotropic polypeptide (GIP), by inhibiting the activity of DPP-4 (19). GLP-1 and GIP stimulate insulin secretion and suppress glucagon release in the gastrointestinal tract, aiding in the maintenance of blood sugar levels (20). Consequently, the use of vildagliptin is associated with reducing blood glucose variability, contributing to improved glycemic control in patients.

We found that vildagliptin significantly reduced HbA1c and GA levels in patients treated with premixed insulin. At the endpoint of the study, HbA1c levels in the vildagliptin group showed a notable reduction of 1.1%, compared to a 0.6% decrease in the control group. Previous studies in western populations have demonstrated similar outcomes (21), but the more pronounced reduction observed in our study suggests that Asian patients may exhibit heightened responsiveness to vildagliptin (22). Asians tend to have a higher proportion of visceral fat, and even those with normal or low body weight often exhibit higher insulin sensitivity (23, 24), which may contribute to better glycemic control. A study conducted in Japanese patients showed similar findings, with an HbA1c reduction of 1.0% following vildagliptin treatment (25).

Through FGM we observed that after 3 months of treatment with vildagliptin add-on premixed insulin, the hourly mean blood glucose concentration was significantly lower than treatment with premixed insulin alone. Vildagliptin particularly reduced postprandial glucose levels following dinner. Previous studies have similarly found that compared to gliclazide or other diabetes-lowering medications, vildagliptin provides a more pronounced improvement in postprandial glucose control (26, 27). This may be attributed to its beneficial effects on β-cell function, which helps in achieving better overall glycemic control throughout the day (28, 29). In our study, the addition of vildagliptin was associated with reduced 24-hour glucose variability. Similar results have been observed in comparisons between vildagliptin and sitagliptin, where vildagliptin demonstrated a lower MAGE and reduced postprandial glucose levels (30), contributing to improved overall glucose management.

After treatment with vildagliptin, a significant reduction in the daily insulin dosage was observed. This aligns with previous studies that have reported decreased daily insulin requirements in patients receiving combined vildagliptin and insulin therapy (31). The reduction was particularly pronounced in patients who had been on high insulin doses for many years (32). A lower insulin dosage reduces the risk of hypoglycemia and mitigates weight gain associated with high insulin intake (33). Additionally, decreasing insulin requirements alleviates the treatment burden on patients, enhances their quality of life, improves adherence to therapy, and contributes to better overall treatment outcomes (34).

Previous studies have demonstrated that vildagliptin exhibits both strong efficacy and safety, particularly by reducing the incidence of hypoglycemic events when used as monotherapy (35, 36). However, due to the insulin-sensitizing effects of DPP-4 inhibitors and the inherent risk of hypoglycemia associated with premixed insulin (6, 37), we hypothesize that the combination of vildagliptin and premixed insulin may lead to an increased likelihood of hypoglycemic episodes. In this study, we observed a significant increase in TBR in the vildagliptin group compared to the control group, which has seldom been reported in prior research on the combination of vildagliptin with insulin. In the study by Ippei Kanazawa et al., the number of patients experiencing three or more hypoglycemic episodes per year was significantly lower in the vildagliptin group than in the control group (38). However, their study did not use FGM for continuous 24-hour glucose monitoring, potentially overlooking asymptomatic hypoglycemia. FGM provides a more comprehensive depiction of daily glucose fluctuations (39), suggesting that when treating with the combination of vildagliptin and premixed insulin, clinicians should be particularly vigilant for potential hypoglycemic events. Furthermore, additional research is required to determine whether the hypoglycemic risk associated with vildagliptin is a widespread phenomenon in combination therapies.

Our study has certain design limitations that should be acknowledged. First, the modest sample size may limit the generalizability of our findings, particularly given the predominance of patients using premixed insulin in our cohort. Secondly, the relatively short study duration of three months prevents us from assessing the long-term impact of vildagliptin on diabetes-related complications. Since improved glycemic variability has been associated with a reduced risk of complications, future studies with extended follow-up periods are warranted to determine whether the observed improvements in glucose stability translate into lasting clinical benefits.

In conclusion, vildagliptin effectively reduces mean blood glucose levels and decreases glycemic variability in patients with T2DM. However, caution is warranted regarding the potential hypoglycemia associated with vildagliptin.

## Data Availability

The original contributions presented in the study are included in the article/supplementary material. Further inquiries can be directed to the corresponding authors.
